# Clinical Predictive Models for Chemotherapy-Induced Febrile Neutropenia in Breast Cancer Patients: A Validation Study

**DOI:** 10.1371/journal.pone.0096413

**Published:** 2014-06-19

**Authors:** Kai Chen, Xiaolan Zhang, Heran Deng, Liling Zhu, Fengxi Su, Weijuan Jia, Xiaogeng Deng

**Affiliations:** 1 Breast Tumor Center, Sun Yat-sen Memorial Hospital, Sun Yat-sen University, Guangzhou, P.R. China; 2 Department of Pediatric Surgery, Sun Yat-sen Memorial Hospital, Sun Yat-sen University, Guangzhou, P.R. China; University of Toronto, Canada

## Abstract

**Background:**

Predictive models for febrile neutropenia (FN) would be informative for physicians in clinical decision making. This study aims to validate a predictive model (Jenkin’s model) that comprises pretreatment hematological parameters in early-stage breast cancer patients.

**Patients and Methods:**

A total of 428 breast cancer patients who received neoadjuvant/adjuvant chemotherapy without any prophylactic use of colony-stimulating factor were included. Pretreatment absolute neutrophil counts (ANC) and absolute lymphocyte counts (ALC) were used by the Jenkin’s model to assess the risk of FN. In addition, we modified the threshold of Jenkin’s model and generated Model-A and B. We also developed Model-C by incorporating the absolute monocyte count (AMC) as a predictor into Model-A. The rates of FN in the 1st chemotherapy cycle were calculated. A valid model should be able to significantly identify high-risk subgroup of patients with FN rate >20%.

**Results:**

Jenkin’s model (Predicted as high-risk when ANC≦3.1*10∧9/L;ALC≦1.5*10∧9/L) did not identify any subgroups with significantly high risk (>20%) of FN in our population, even if we used different thresholds in Model-A(ANC≦4.4*10∧9/L;ALC≦2.1*10∧9/L) or B(ANC≦3.8*10∧9/L;ALC≦1.8*10∧9/L). However, with AMC added as an additional predictor, Model-C(ANC≦4.4*10∧9/L;ALC≦2.1*10∧9/L; AMC≦0.28*10∧9/L) identified a subgroup of patients with a significantly high risk of FN (23.1%).

**Conclusions:**

In our population, Jenkin’s model, cannot accurately identify patients with a significant risk of FN. The threshold should be changed and the AMC should be incorporated as a predictor, to have excellent predictive ability.

## Introduction

Febrile neutropenia (FN) is one of the most common complications in breast cancer patients treated with chemotherapy. Approximately 25–40% of treatment-naïve patients develop FN [1]. FN may predispose patients to life-threatening infection and/or broad-spectrum antibiotic use, prolonged hospitalization, treatment delay or dose reductions[2]. Therefore, prophylactic use of colony stimulating-factor (CSF) in selected patients is critical. Many guidelines recommend that the decision to use CSF prophylactically should depend on the risk of FN with the chemotherapy regimens[3]–[8], which have been categorized into high-risk (>20%), intermediate-risk (10–20%) and low-risk (<20%) regimens of FN.

Although the chemotherapy regimen is the most critical external reason for FN in breast cancer patients, it should not be ignored that even for those patients receiving dose-dense chemotherapy regimens, 30–50% of them will not experience FN [9]–[11]. Therefore, internal reasons exist that may account for FN. Advanced or metastatic disease, age, comorbidity status, history of some chronic diseases, liver function and renal function have all been reported to be associated with FN[12]–[17]. These factors, however, are not a direct reflection of the granulocyte reservoir or the stem cell pool of the bone marrow. Therefore, pretreatment hematological parameters, such as white blood cell count[18], platelet count[19], absolute neutrophil count (ANC) [20], [21], absolute lymphocyte count (ALC) [22], [23] or absolute monocyte count (AMC) [16], [19], [24], are hypothesized to reflect, to some extent, the patient’s predisposition to FN. Jenkins et al. developed a model using pretreatment ANC and ALC in breast cancer patients receiving CEF (5-fluorouracil, epirubicin and cyclophosphamide) chemotherapy[20]. They categorized patients into five subgroups based on different combinations of quintiles of ANC and ALC values [20], [21]. Group V (ANC≤3.1×10^9^/L & ALC≤1.5×10^9^/L) was defined as a high-risk subgroup in their studies with an FN risk higher than 20%. Their model has been externally validated in breast cancer patients receiving the TAC (docetaxel, adriamycin and cyclophosphamide) regimen, which showed a high risk of FN (>20%) [21].

The aims of this study are 1) to evaluate whether the pretreatment hematological parameters are predictive of FN and 2) to validate Jenkin’s predictive model in our population.

## Methods

### Patients and Data Collection

We searched our database for early-stage breast cancer patients who received neoadjuvant/adjuvant chemotherapy between 2005 and 2013 at our Sun Yat-sen Memorial Hospital. Exclusion criteria include 1) stage IV breast cancer, 2) history of other cancers, 3) essential data unavailable, 4) history of anemia or other hematological disorders, 5) the first chemotherapy cycle was not administered at our hospital, and 6) prophylactic use of CSF. A total of 428 patients were finally identified and included. All of the included patients received breast-conserving surgery or mastectomy when appropriate. FN was defined as a temperature >38.5°C and an ANC<0.5×10^9^/L or<1.0×10^9^/L and expected to fall below 0.5×10^9^/L. In the current study, we only focused on FN occurring in the 1^st^ cycle of chemotherapy. Based on the policy of our institution, we did not administer prophylactic CSF for chemotherapy in early-stage breast cancer patients, except for those who received dose-dense regimens. Clinicopathological features of the patients and the results of the hematological tests were extracted from the medical records. For patients with no FN events recorded in our database, we performed telephone interviews for confirmation. This study was approved by the Institutional Review Broad of Sun Yat-sen Memorial Hospital. Written informed consents were obtained from the included patients.

### Chemotherapy

The chemotherapy regimens were employed as follows: CMF, cyclophosphamide + methotrexate+5-fluouracil; EC, epirubicin + cyclophosphamide; TC, paclitaxel + cyclophosphamide; DC, docetaxel + cyclophosphamide; CEF, cyclophosphamide + epirubicin+5-fluouracil; ET, epirubicin + paclitaxel; TEC, epirubicin + paclitaxel+ cyclophosphamide; ED, epirubicin + docetaxel; and DEC, epirubicin + docetaxel + cyclophosphamide. Patients were required to have whole blood counts measured at baseline, as well as on the 7^th^, 9^th^ and 14^th^ days of each chemotherapy cycle, and the results and/or any febrile events were reported to their physician. CSF (filgrastim 5 mcg/kg until post-nadir ANC recovery) was employed for ANC <1.0×10^9^/L at the 7^th^ or 9^th^ day of each cycle. Antibiotics were employed at any time when FN occurred. No prophylactic antibiotics were used before treatment.

### Statistical Consideration

For the comparison of FN rates in patients with different pathological features, Fisher’s exact test/chi-squared test and the Mann-Whitney U test were used for categorical and continuous variables, respectively. The Mann-Whitney U test was also used in univariate analysis to screen the pretreatment blood count variables for independent risk factors of FN.

In the Jenkin’s model, patients were classified into five subgroups (Group I–V) based on their ANC and ALC values [21], [25]. To validate the Jenkin’s model, we calculated the FN rate of each subgroup (Table 1). The model was considered valid if the actual rate of FN in the predicted high-risk group (Group V) was higher than 20% and the FN rates among subgroups were of statistical significance. In addition, we modified Jenkin’s model by combining the five subgroups into two subgroups (low-risk and high-risk). The modified Jenkin’s models A and B (referred to as Model-A and Model-B hereafter) were generated as follows:

**Table 1:** Patients features of the included patients.

Items		n		FN		no-FN		P
				n	%	n	%	
Age (yrs, Mean±std)		47.3±9.8		45.9±9.3		47.5±9.9		NS
BMI (Mean±std)		23.2±4.0		22.7±3.8		23.3±4.0		NS
BSA (m∧2 Mean±std)		1.6±0.2		1.52±0.2		1.56±0.2		NS
Hypertension history								
No		386		54	14	332		<0.05
Yes		41		1	2	40	98	
Diabetes history								
No		411		53	13	358		NS
Yes		17		2	12	15	88	
Menopausal status								
Pre/peri-menopausal status		276		38	14	238		NS
Post menopausal status		146		15	10	131	90	
T-stage								
T1		196		22	11	174		NS
T2		214		31	14	183	86	
T3		12		2	17	10	83	
N-stage								
N0		277		28	10	249		<0.05
N1		95		17	18	78	82	
N2		32		4	13	28	88	
N3		22		6	27	16	73	
Pathology subtype								
IDC		376		15	4	361		NS
ILC		7		1	14	6	86	
IDC+ILC		12		1	8	11	92	
Others		33		2	6	31	94	
ER status								
Negative		81		12	15	69		NS
Positive		340		43	13	297	87	
PR status								
Negative		121		12	10	109		NS
Positive		301		43	14	258	86	
HER2								
Negative		292		34	12	258		<0.05
Intermediate		31		1	3	30	97	
Positive		104		20	19	84	81	
Ki67								
Negative		101		11	11	90		NS
Positive		303		39	13	264	87	
Neoadjuvant chemotherapy								
No		98		20	20	78		<0.05
Yes		328		35	11	293	89	
Blood type								
A		120		12	10	108		NS
B		108		20	19	88	81	
O		172		20	12	152	88	
AB		22		2	9	20	91	
Chemotherapy regimens?								
CMF		18		0	0	18		<0.01
CEF		21		1	5	20	95	
EC		22		5	23	17	77	
TC		53		1	2	52	98	
DC		29		2	7	27	93	
TEC		16		3	19	13	81	
DEC		43		14	33	29	67	
ET		78		11	14	67	86	
ED		62		17	27	45	73	
Others		7		1	14	6	86	
Chemotherapy regimens with different risk of FN?.								
Low	222		9		4	213		<0.01
Intermediate	101		15		15	86	85	
High	105		31		30	74	70	

BMI, body mass index. BSA, body surface area ER, estrogen receptor; PR, progesterone receptor; HER2, human epidermal growth factor receptor 2; FN, febrile neutropenia.?High risk regimen(DEC, DE); Intermediate risk regimen (TEC,ET OTHERS); ?C, Cyclophosphomide;M, Methotrexate; E, epirubicin; F, 5-Fluorouracil; T, Paclitaxle; D, Docetaxel;Others included regimens contained herceptins cisplatin, or nolvelbine; NS, non-significant;

#### Model-A

Group I and II as a low-risk subgroup; Group III, IV and V as a high-risk subgroup.

#### Model-B

Group I, II and III as a low-risk subgroup; Group IV and V as a high-risk subgroup.

To improve the performance of Model-A, we incorporated the AMC value as one of the predictors and generated Model-C. Patients in the high-risk subgroup of Model-A were classified as high-risk in Model-C when their AMC values were lower than a specific threshold. To determine the optimal threshold of AMC for Model-C, we used ROC curves and calculated the corresponding AUC and P values when a different threshold of AMC was used. The AMC value that enabled the AUC and P value of Model-C to reach a significant level was used as the threshold of AMC. Multivariate analysis was performed using logistic regression. All tests of significance were two tailed. Statistical analyses were carried out using SPSS v18.0 (Chicago, IL, USA).

## Results

### Clinicopathological Features

**Figure 1:**
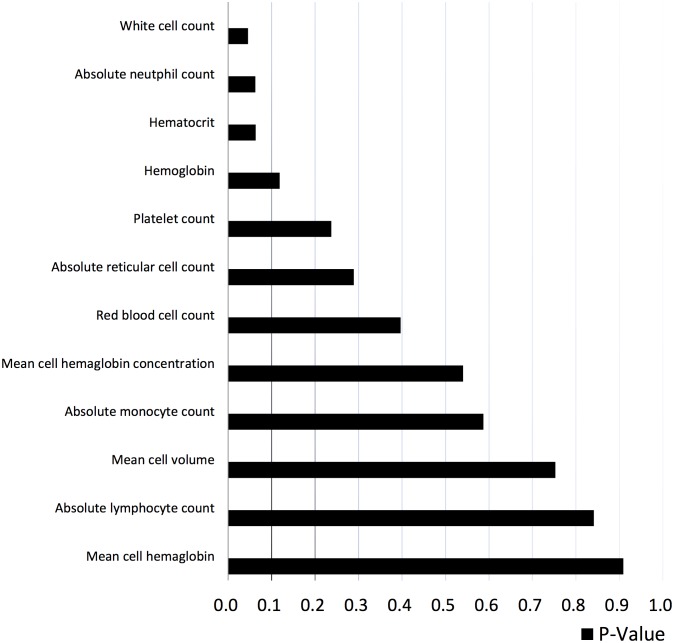
Univariate analysis of predictive hematological factors for FN. Mann-Whitney U test was used as a univariate analysis and the P-value was shown. White cell count, absolute neutrophil count and hematocrit with P-value less than 0.1 was incorporated into multivariate analysis.

The clinicopathological features and hematological test results of the 428 patients are summarized in Table 1 and Figure 1. The mean and median pretreatment WBC, ANC and ALC values of our population are comparable to those in Jenkin’s studies (Table 2). Fifty-five patients (12.8%) developed FN during the 1^st^ cycle of chemotherapy. The median and mean ANC nadir in FN patients was 0.06×10^9^/L and 0.20×10^9^/L, respectively.

**Table 2:** Pretreatment WBC, ANC and ALC values in our pupulation are comparable to Jenkins study.

Items	2009 Dataset in Jenkin’s study		Our dataset		Our dataset			
	Mean±SD(10∧9/L)	Normal range (10∧9/L)	Mean±SD(10∧9/L)	Normal range (10∧9/L)	Median (10∧9/L)	Range (10∧9/L)	Median (10∧9/L)	Range (10∧9/L)
WBC	6.96±1.82	3.60–11.00	6.70±2.00	4.00–10.00	6.90	3.80–19.5	6.46	2.39–13.47
ANC	4.32±1.48	1.80–7.50	4.30±1.80	2.00–7.50	4.30	1.60–17.9	3.93	0.35–12.47
ALC	2.02±0.64	1.50–4.00	1.90±0.60	0.80–4.00	1.90	0.30–4.4	1.82	0.07–4.63

### Univariate Analysis of Clinicopathological Factors and Pretreatment Hematological Parameters

**Figure 2:**
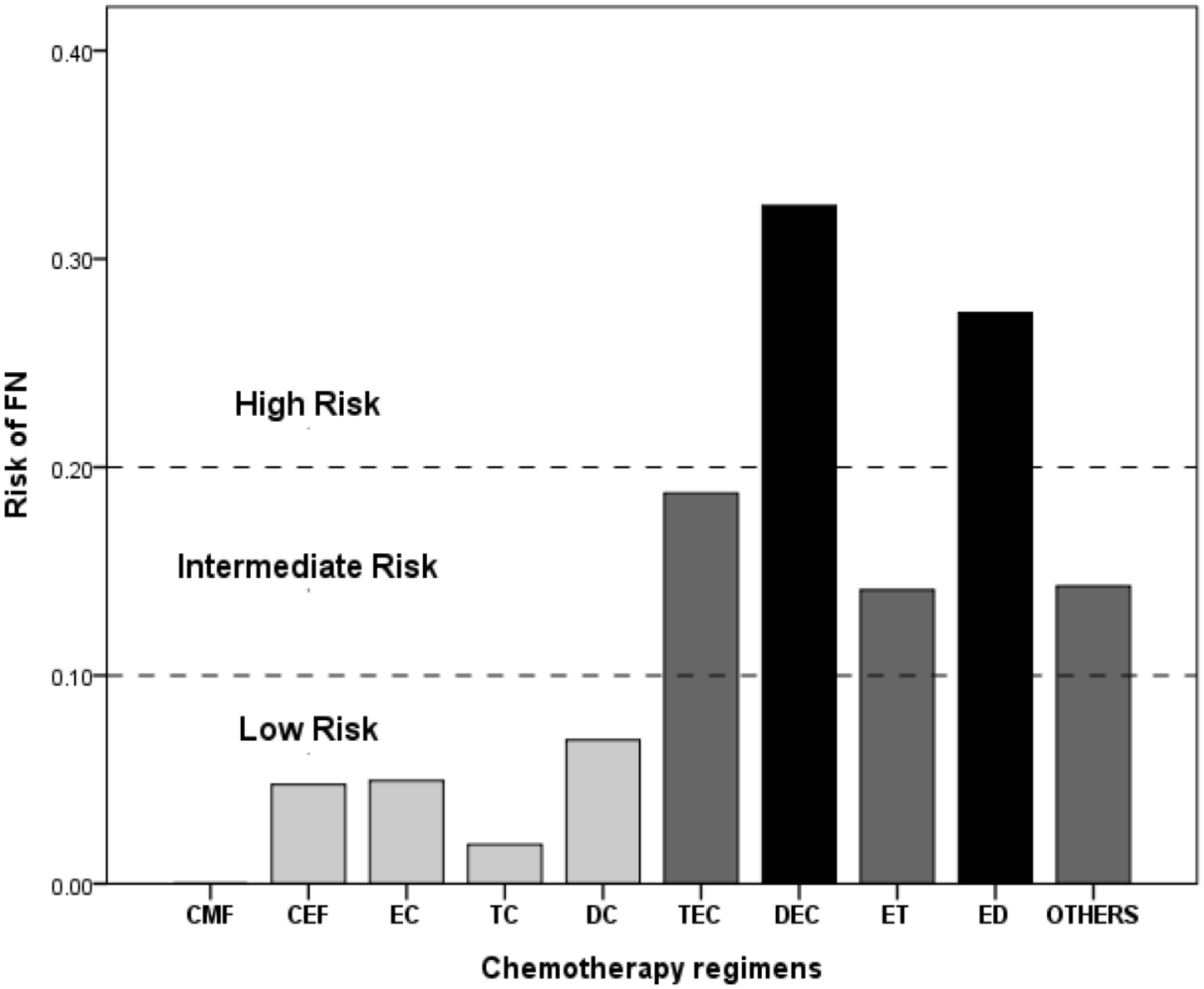
FN rate in patients receiving different chemotherapy regimens. Chemotherapy regimens were catagorized into high-, intermediate- or low-risk regimens based on their probability of having FN events.

History of hypertension (P<0.05), N stage (P<0.05), Her2 status (P<0.05), neoadjuvant chemotherapy (P<0.05), white cell count (P<0.05) and chemotherapy regimens (P<0.01) were significantly associated with FN. Hematocrit (P = 0.06) and ANC (P = 0.06) were marginally significant in predicting FN. The FN rates of different regimens are shown in Figure 2. DEC and ED were classified as high-risk regimens (>20%), whereas TEC, ET and others (carboplatin- and/or trastuzumab-based regimens) were classified as intermediate-risk regimens (10–20%). CMF, CEF, EC, ET, TC and DC regimens were classified as low risk (<10%).

### Validation of Jenkin’s Model

Jenkin’s model classified patients into five subgroups. The number of patients distributed in these subgroups in our dataset is similar to that reported by Jenkin et al. in 2009[20] and 2012[21] (see Figure S1 and Table S1). Based on Jenkin’s model, the FN rates were not significantly different among the five subgroups in our patients, and none of them had an FN rate higher than 20% (Table 3). Model-A, rather than Model-B, could identify patients with a significantly higher FN rate (17.2% vs. 9.7, P<0.05), but did not reach the 20% high-risk threshold.

**Table 3:** Validation of Jenkin’s model and Modified Jenkin’s model.

Group*	ANC (×10∧9/L)	ALC (×10∧9/L)	AMC (×10∧9/L)	Total No.	FN in the 1st cycle		P†
					No.	%	
Jenkin’s model							
Group I	>5.2	>2.4	n/a	155	15	9.7	NS
Group II	≦5.2	≦2.4	n/a	93	9	9.7	
Group III	≦4.4	≦2.1	n/a	72	14	19.4	
Group IV	≦3.8	≦1.8	n/a	69	13	18.8	
Group V (High-risk subgroup)	≦3.1	≦1.5	n/a	39	4	10.3	
Model-A							
Low-risk subgroup (Group I & II)	>4.4	>2.1	n/a	248	24	9.7	<0.05
High-risk subgroup (Group III,IV & V)	≦4.4	≦2.1	n/a	180	31	17.2	
Model-B							
Low-risk subgroup (Group I,II & III)	>3.8	>1.8	n/a	320	38	11.9	NS
High-risk subgroup(Group IV & V)	≦3.8	≦1.8	n/a	108	17	15.7	
Model-C							
Low-risk subgroup	Not fulfill the criteria of high-risk group			337	34	10.1	<0.01
High-risk subgroup	≦4.4	≦2.1	≦0.28	91	21	23.1	

*In Jenkin’s model, patients were classified into different groups without overlaps. For example, group IV comprises patients with ANC ≦3.8 and ALC ≦1.8 who do not fulfil the criteria for group V.

† Chi-square test was used.

**Figure 3:**
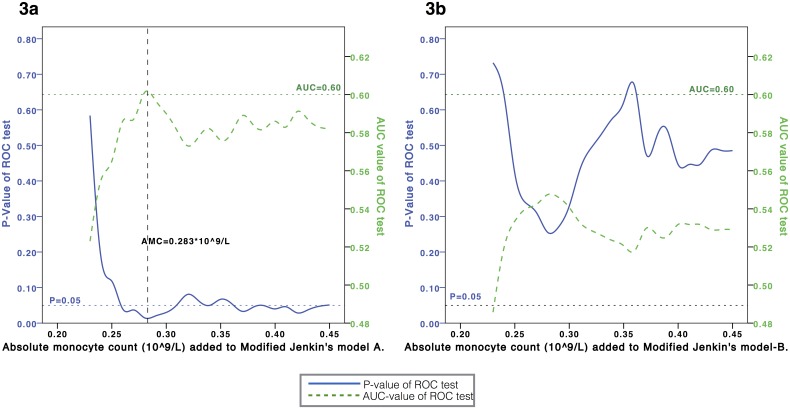
Optimal threshold of AMC. To incorporate AMC into Model-A (3a) or Model-B (3b), we calculated the AUC and P-value of the new model when different threshold of AMC was used. A new model (Model-C) could be developd from Model-A (3a) with the highest AUC value and lowest P value, when the threshold of AMC = 0.283*10∧9/L. No valid model could be established when AMC was incorporated into Model-B (3b).

Therefore, we investigated whether incorporating the AMC value could improve the performance. As shown in Figure 3a, the performance of Model-A can reach a plateau with AUC≈0.58–0.60 and P≈0.05 for an AMC threshold value>0.28×10^9^/L. By contrast, the performance of Model-B could not be improved regardless of the AMC value used (Figure 3b). Therefore, the optimal threshold of AMC to be used should be 0.28×10^9^/L, and a new model (Model-C) was generated based on Model-A:

#### Model-C

High-risk subgroup: ANC≤4.4×10^9^/L, ALC≤2.1×10^9^/L and AMC≤0.28×10^9^/L.

Low-risk subgroup: Patients do not fulfill the criteria for inclusion in the high-risk subgroup.

The high-risk subgroup in Model-C demonstrated a significantly higher FN rate compared with the low-risk subgroup (23.1% vs. 10.1%; P<0.01). The sensitivity, specificity, false-negative rate, false-positive rate, positive predictive value and negative predictive value were 38.2%, 81.2%, 61.8%, 18.8%, 23.1% and 89.1%, respectively.

### Multivariate Analysis

Clinicopathological factors and pretreatment hematological factors that were shown to be associated with FN in the univariate analysis, together with the chemotherapy regimen (classified as low-, intermediate- and high-risk) and Model-C (low-risk vs. high-risk subgroup), were included in logistic regression as the multivariate analysis. The chemotherapy regimen (intermediate- vs. low-risk regimen (HR = 3.51; P<0.01; 95% CI: 1.45–8.53); high- vs. low-risk regimen (HR = 9.48; P<0.01; 95% CI: 4.26–21.1)) and the Model-C subgroup (high- vs. low-risk group; HR = 2.77; P<0.01; 95% CI: 1.42–5.37) are the only two independent predictors for FN.

## Discussion

### Chemotherapy Regimens and FN

Assessing the risk of FN would be informative for physicians in clinical decision making before chemotherapy. The regimens and dosage are the major considerations when evaluating the risk of FN. The estimated risk of FN from each regimen suggested by the current guidelines was limited by the specific populations, study methods and different clinical scenarios. For example, the CMF (cyclophosphamide, methotrexate, fluorouracil) regimen is classified as a low-risk (<10%) or intermediate-risk (10–20%) regimen in the EORTC [3] or NCCN [5] guidelines, respectively. Hence, we assessed the FN rate in different chemotherapy regimens in our population. Consistent with the NCCN and EORTC guidelines, the FN rate of our DAC regimen was higher than 20%. When paclitaxel, instead of docetaxel, was used in combination with anthracycline +/− cyclophosphamide, the FN rate fell into the 10–20% range. In the NCCN guidelines, docetaxel every 21 days and CMF regimens are considered intermediate-risk regimens. However, in our population, these two regimens had a low risk of FN (<10%)[5], consistent with the EORTC guidelines [3]. Therefore, physicians should summarize the FN rate of each regimen in their own population to gain reliable reference information for clinical decision making. Applying any of the guidelines without prior validation is not appropriate.

### Validation of Jenkin’s Model in the Population

Developing a predictive model for FN is important. In patients who receive high-risk regimens with the support of prophylactic CSF, an accurate model may enable the identification of those who may still have FN and the subsequent dose deduction. Patients could also be well informed about the possible complications. A predictive model could also be helpful for patients with an intermediate risk of FN (10–20%) when the use of prophylactic CSF is determined by the physician. Several models have been developed and widely validated in cancer patients[9], [14], [16], [19], [22], [24]. However, few models have been developed specifically for breast cancer patients. The INC-EU (Impact of Neutropenia in Chemotherapy European study group) reported a multivariate model in breast cancer patients[15], but they did not present it as an applicable formula or nomogram for external validation. In the present study, we tested whether Jenkin’s model is valid in our patients. Prior to that, we screened our pretreatment hematological parameters and found that only the ANC was marginally associated with FN status. ANC, ALC or AMC alone was not associated with FN. Similar findings were also observed in one of Jenkin’s studies, in which the ANC and ALC were not by themselves correlated with the frequency of FN. However, their patients, when combined into five groups based on the Jenkin’s model, had significant differences in the risk of FN in any cycle or in the 1^st^ cycle[21]. When testing the Jenkin’s model in our population, we noticed that group V patients (ANC≤3.1×10^9^/L & ALC≤1.5×10^9^/L), who are defined as a high-risk subgroup in the Jenkin’s model, did not have an FN rate higher than 20%. The following explanations for the failure of the Jenkin’s model were considered:

1. The distribution of baseline hematological parameters differed among our population and Jenkin’s. This explanation could be ruled out because we compared the mean and median values of the WBC, ANC and ALC in our populations with those in the Jenkin’s studies and did not observe any significant differences (Table 2). In addition, the number of the patients distributed in the different subgroups was also similar among the populations (see Figure S1 and Table S1).
2. The FN rate among our populations (12.8%) and those in Jenkin’s studies are different (8% and 6% in the 2009 and 2012 studies, respectively). In addition, Jenkin et al. used the same regimen (CEF in the 2009 study and TEC in the 2012 study) in their population, whereas different chemotherapy regimens were used in our patients. These might be the most likely reasons that cannot be ruled out. Our study did not have a sufficiently large sample size to validate Jenkin’s model in patients receiving the same chemotherapy regimens.
3. In Jenkin’s model, they considered patients in Group V to be the high-risk subgroup. Because Group V patients did not have a significant higher risk of FN in our populations, we tried to use different thresholds of Jenkin’s model by combining the five subgroups into two and generated Model-A and -B (described in the Methods and Results sections). As shown in Table 3, Model-A and -B did not perform well either.

Taken together, our data suggested that the Jenkin’s model may not be valid in our population.

### Incorporation of AMC into the Jenkin’s Model

To improve the Jenkin’s model, we incorporated the AMC as a predictor based on our hypothesis that the combination of ANC, ALC and AMC could comprehensively reflect the bone marrow granulocyte reservoir and, therefore, predict the chemotherapy-induced FN. Kondo et al. and Oguz et al. reported that an AMC<0.15×10^9^/L was an independent factor for FN in solid tumors[13], [24]. With the same threshold, Moreau’s study also suggested that the baseline AMC could independently predict FN in hematological malignancies[26]. In our study, the quintile values of AMC were 0.23×10^9^/L, 0.30×10^9^/L, 0.36×10^9^/L and 0.45×10^9^/L. There were only 16 (3.7%) patients with a pretreatment AMC<0.15×10^9^/L, and none of them had FN events. Therefore, an AMC<0.15×10^9^/L may not be an optimal threshold. Our study revealed that the AMC threshold should be higher than 0.28×10^9^/L to enable the AUC to reach a significant level (Figure 3). We applied 0.28×10^9^/L as the AMC threshold in Model-C, which identified patients with a significantly high risk of FN (23%). This result is very surprising because AMC only constitutes a small percentage of the WBC but plays such a critical role in risk assessment. Model-C might be able to reflect the patients’ internal reasons that determine their predisposition to FN. In addition, the predictive ability of Model-C was independent of the chemotherapy regimens, as shown by our multivariate analysis. Therefore, to comprehensively evaluate the risk of FN, we propose that the pretreatment ANC, ALC and AMC values should all be considered, in addition to the chemotherapy regimens.

All of the patients received surgical treatment in our study. However, it is unknown whether the sequence of chemotherapy and surgery would have any influences on the model predicting accuracy. As a confounding factor, neoadjuvant chemotherapy was associated with FN in univariate analysis, but not in multivariate analysis, suggesting that the sequence of chemotherapy and surgery was not independently associated with FN. In multivariate analysis, we also assessed but did not observe any interaction between neoadjuvant chemotherapy and Model-C, which indicated that the surgical treatment or not would have no impact on the model prediction accuracy in this study.

### Limitations of our Study

Several limitations of our study should be addressed.

1. We only focused on the FN that occurred during the 1^st^ cycle of chemotherapy. We are uncertain whether our model can be predictive for FN occurring for the duration of chemotherapy. However, because approximately 80% of FN occurred during the 1^st^ cycle of thermotherapy, our study may still be valid for testing the predictive models. To predict the risk of FN occurred in the 2^nd^ cycle of chemotherapy or later, more “post-chemo” hematological parameters could be incorporated to improve the model performance.
2. The sample size of our population was not sufficiently large to assess the performance of the models in each chemotherapy regimen. The dosage of chemotherapy regimen might also have influences on model prediction, which could not be assessed in this study. In addition, we had no patients who received dose-dense chemotherapy, which is presently widely used. However, the multivariate analysis in our study suggested that Model-C is an independent predictor of FN when adjusted for the chemotherapy regimens. Thus, we believe that our Model-C, with AMC as one of the predictors, could predict the patient’s predisposition for FN regardless of the chemotherapy regimens.
3. Chia et al.[17] had studied the association between chronic comorbid condition and the risk of FN and showed that congestive heart failure, osteoarthritis, previous cancer and thyroid disorder were associated with increased risk of FN. In addition, the pretreatment renal function, liver function and chemotherapy dosage, which were shown to be associated with FN, were not included in this study. Therefore, it remains unknown whether these factors may affect the performance of the models in our study.
4. We do not have an external dataset to validate our Model-C.

### Conclusions

In summary, our study suggested that 1) the FN rate of each chemotherapy regimen should be evaluated prior to following any guidelines on the prophylactic use of CSF. 2) The chemotherapy regimen is critical as an external factor when assessing the risk of FN in breast cancer patients. Hematological parameters alone cannot predict FN in our population. 3) Jenkin’s model did not pass the validation test in our populations. 4) Modification of Jenkin’s model with AMC incorporated as a predictor to create a new model (Model-C) was developed with excellent predictive capability.

Further investigations, including external validation of our new model, are needed. Can our model be used to predict the FN rate for the entire duration of chemotherapy or in metastatic breast cancer patients? Can additional parameters, such as indexes of the liver or renal function, be incorporated to improve our model? Can our model be used in patients receiving dose-dense chemotherapy with prophylactic CSF support? Are there any differences of the performance of our model when used in patients with different regimens of chemotherapy? Future studies are needed to help clarify these issues.

## Supporting Information

Figure S1 **Comparison of the population distribution patterns between our dataset and those of Jenkin’s.** Figure S1 suggested that the population distribution patterns were similar between our datasets and those of Jenkin’s.
(TIFF)

Table S1 **Distribution of total patients in different risk groups.**
(DOCX)
