# Supplementary figures and images for: Clinical Predictive Models for Chemotherapy-Induced Febrile Neutropenia in Breast Cancer Patients: A Validation Study
Source: PLoS One. 2014 Jun 19;9(6):e96413. doi: 10.1371/journal.pone.0096413 (PMC4063732; doi:10.1371/journal.pone.0096413)

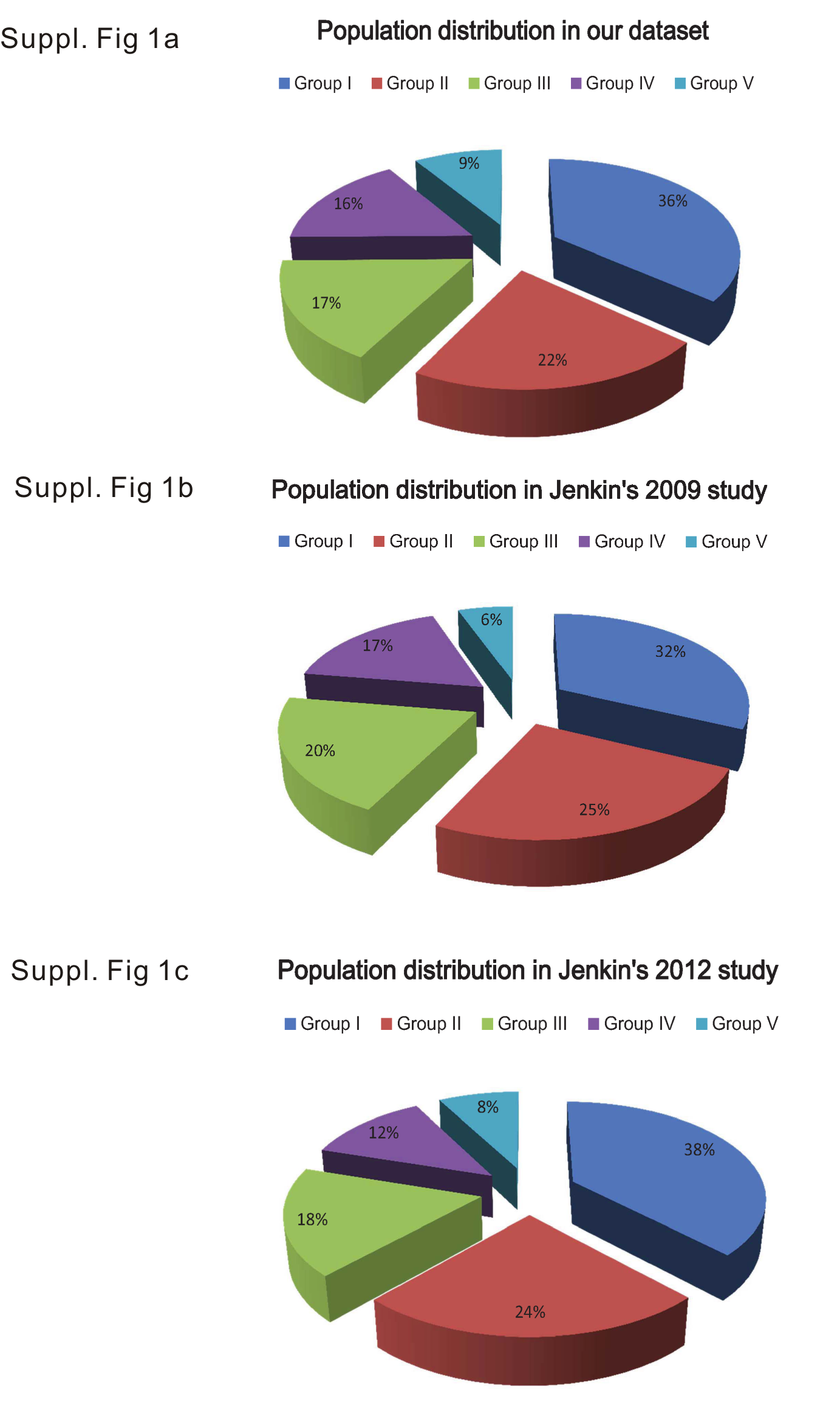

Supplement: Figure S1 — Comparison of the population distribution patterns between our dataset and those of Jenkin’s. Figure S1 suggested that the population distribution patterns were similar between our datasets and those of Jenkin’s. (TIFF) [file pone.0096413.s001.tiff]
