# Supplementary material for: Clinical Predictive Models for Chemotherapy-Induced Febrile Neutropenia in Breast Cancer Patients: A Validation Study
Source: PLoS One. 2014 Jun 19;9(6):e96413. doi: 10.1371/journal.pone.0096413 (PMC4063732; doi:10.1371/journal.pone.0096413)
Supplement: Table S1 — Distribution of total patients in different risk groups. (DOCX) [file pone.0096413.s002.docx]

| Suppl. Table 1, Distribution of total patients in different risk groups | | | | | | |
| --- | --- | --- | --- | --- | --- | --- |
| Group† | Our data set (428 pts) | | 2009 Jenkin's study dataset (=600 pts) | | 2012 Jenkin's study dataset (263 pts) | |
|  | No. | % | No. | % | No. | % |
| Group I | 155 | 36.2 | 192 | 32.0 | 99 | 37.6 |
| Group II | 93 | 21.7 | 152 | 25.3 | 64 | 24.3 |
| Group III | 72 | 16.8 | 121 | 20.2 | 47 | 17.9 |
| Group IV | 69 | 16.1 | 102 | 17.0 | 32 | 12.2 |
| Group V | 39 | 9.1 | 33 | 5.5 | 21 | 8.0 |
| †Groups classified based on Jenkin's model as described in the Method section. | | | | | | |
